# Therapeutic Role of HPV Vaccination on Benign HPV-induced Epithelial Proliferations in Immunocompetent and Immunocompromised Patients: Case Study and Review of the Literature

**DOI:** 10.1093/ofid/ofae369

**Published:** 2024-07-19

**Authors:** Caterina Matucci-Cerinic, Astrid Herzum, Giulia Ciccarese, Silvia Rosina, Roberta Caorsi, Marco Gattorno, Corrado Occella, Gianmaria Viglizzo, Stefano Volpi

**Affiliations:** DINOGMI, University of Genoa, Genoa, Italy; UOC Rheumatology and Autoinflammatory diseases, IRCCS Istituto Giannina Gaslini, Genoa, Italy; UOC Dermatology and Angioma Center, IRCCS Istituto Giannina Gaslini, Genoa, Italy; UOC Dermatologia e Venereologia, Dipartimento di Scienze Mediche e Chirugiche, Università degli Studi di Foggia e Policlinico Riuniti, Foggia, Italy; UOC Rheumatology and Autoinflammatory diseases, IRCCS Istituto Giannina Gaslini, Genoa, Italy; DINOGMI, University of Genoa, Genoa, Italy; UOC Rheumatology and Autoinflammatory diseases, IRCCS Istituto Giannina Gaslini, Genoa, Italy; UOC Rheumatology and Autoinflammatory diseases, IRCCS Istituto Giannina Gaslini, Genoa, Italy; UOC Dermatology and Angioma Center, IRCCS Istituto Giannina Gaslini, Genoa, Italy; UOC Dermatology and Angioma Center, IRCCS Istituto Giannina Gaslini, Genoa, Italy; DINOGMI, University of Genoa, Genoa, Italy; UOC Rheumatology and Autoinflammatory diseases, IRCCS Istituto Giannina Gaslini, Genoa, Italy

**Keywords:** HPV-vaccination, human papilloma-virus, immunosuppressed, vaccine, warts

## Abstract

Human papillomavirus (HPV) vaccination represents a milestone in primary prevention of sexually transmitted infections. However, little is known about its possible effects on already established HPV infections. We report the case of a 9-year-old immunosuppressed girl with refractory warts, successfully treated with the nonavalent-HPV vaccine and review the literature about the therapeutic effects of HPV vaccination on benign HPV-induced epithelial proliferations in immunocompetent and immunosuppressed patients. In the literature, promising results were shown on cutaneous warts after HPV vaccination, especially in children and young adults, also in immunosuppressed patients, whereas controverse results were found on anogenital warts. These findings suggest a critical need for randomized clinical trials to assess the efficacy of HPV vaccination in the treatment of benign HPV-induced epithelial proliferations.

## Teaser

- HPV vaccine seems effective in treating recalcitrant cutaneous/anogenital warts
- A better response was observed in children than in adults
- In immunosuppressed patients, HPV vaccination could be considered for recalcitrant warts

Human papilloma viruses (HPVs) are common oncogenic double-stranded DNA viruses classified on the basis of their major capsid protein gene (L1) nucleotide sequence, on which their tropism (cutaneous or mucosal) and tumorigenic power depends [1]. More than 100 HPV types have been described and classified according to their oncogenic potential into low-risk (LR) or high-risk (HR) oncogenic subtypes [2]. Clinically, HPV infections can be asymptomatic, or cause epithelial proliferations, resulting in benign cutaneous warts (CW) (mainly because of LR-HPV types 1–4, 26–29) and anogenital warts (AW) (mainly because of LR-HPV types 6, 11), as well as malignant anogenital proliferations (because of HR-HPV types 16, 18, 31, 33, 35, 39, 45, 52, 55, 56, 58) [1, 2]. Among the latter, HPV 16 and 18 account for 70% of all cervical cancers, the fourth most common cancer in women worldwide [3–5].

Because of this, as primary prevention measure to reduce HPV-related tumors, HPV vaccines were developed. Since 2006, the Food and Drug Administration approved the quadrivalent HPV vaccine (4V), that targets HPV types 6, 11, 16, 18; the bivalent HPV vaccine (2V), targeting HR-HPV types 16 and 18, and the nonavalent HPV vaccine (9V), targeting HPV types 6, 11, 16, 18, 31, 33, 45, 52, 58 [6–8]. These vaccines proved able to significantly prevent the occurrence of high-grade cervical intraepithelial neoplasia related to HPV-16 or HPV-18 [9, 10].

HPV vaccines are based on type-specific HPV L1 major capsid protein, assembled to form recombinant, noninfectious virus-like particles (VLPs), antigenically identical to infective virions.

In the past, for recalcitrant CW and AW, many different approaches have been tried. The most widely used are physical treatments such as cryotherapy, chemical cauterization, curettage, electrodessication, and laser removal, with which a recurrence is documented in up to 30% of patients probably because of a lack of immune response [11]. Another approach is represented by immunomodulatory therapies that can be administered systemically, intralesionally, or intradermally and topically and work by stimulating the local immune response against the causative agent, thereby leading to complete resolution and decreased recurrences. Some agents like cimetidine, levamisole, and zinc have been studied in few randomized trials with different results. Among these, also the administration of HPV vaccine is considered to act as an immunomodulatory agent [12].

The VLP-based vaccines have a prophylactic role, eliciting the host's subtype-specific neutralizing antibody response, which inhibits the viral entrance in epithelial cells and might confer also a degree of cross-protection against nonvaccine-specific subtypes [13, 14]. HPV vaccination has also been shown to induce a local cell-mediated immune response involved in the eradication of infections that could provide some efficacy among individuals already infected with HPV [15–20]. Even if a randomized controlled trial [15] conducted on women infected with cervical HPV showed no efficacy of the vaccination in clearing the infection when compared to controls, and another trial conducted on HIV patients did not show an evident benefit of the HPV vaccination on premalignant/dysplastic conditions [21], several reports have shown an efficacy of HPV vaccination in the clearing of benign CW and AW. Currently, HPV therapeutic vaccines are under trial for cervical, anal, and vulvar intraepithelial neoplasia and cervical cancer [22].

In the present work, we present the case of an immunosuppressed 10-year-old girl with recalcitrant CW and AW successfully treated with the 9V and provide a review of the literature regarding the possible therapeutic effects of HPV vaccination on benign HPV-induced epithelial proliferations in immunocompetent and immunosuppressed pediatric and adult patients.

## METHODS

On PubMed, a literature search of English reports on the use of HPV vaccination for the treatment of HPV-induced epithelial proliferations in immunocompetent and immunosuppressed adults and children was performed. To retrieve all relevant articles, the following terms were researched: “HPV,” “human papillomavirus,” “warts,” “HPV-vaccination,” “pediatric,” “treatment,” “immunosuppressed,” “immunodeficiency.” The research was conducted independently by 2 researchers (C.M.C., A.H.). Abstracts were first screened to assess eligibility. Also, literature reviews and included papers’ bibliographies were screened for additional references. The patients were considered pediatric if aged <18 years. Patients in which the efficacy of the vaccination could not be assessed or treated with experimental vaccines were not included. The response to vaccination (complete, partial, absent) was annotated as reported in the studies (in the majority of papers the resolution of all lesions after the treatment was considered as complete resolution, whereas a partial response was considered as a decrease in the size and/or number of more than 50% of target lesions, and an absent response as no modification or appearance of new lesions). For this review, the patients were then divided in immunocompetent and immunosuppressed.

## CASE REPORT

**Figure 1.**
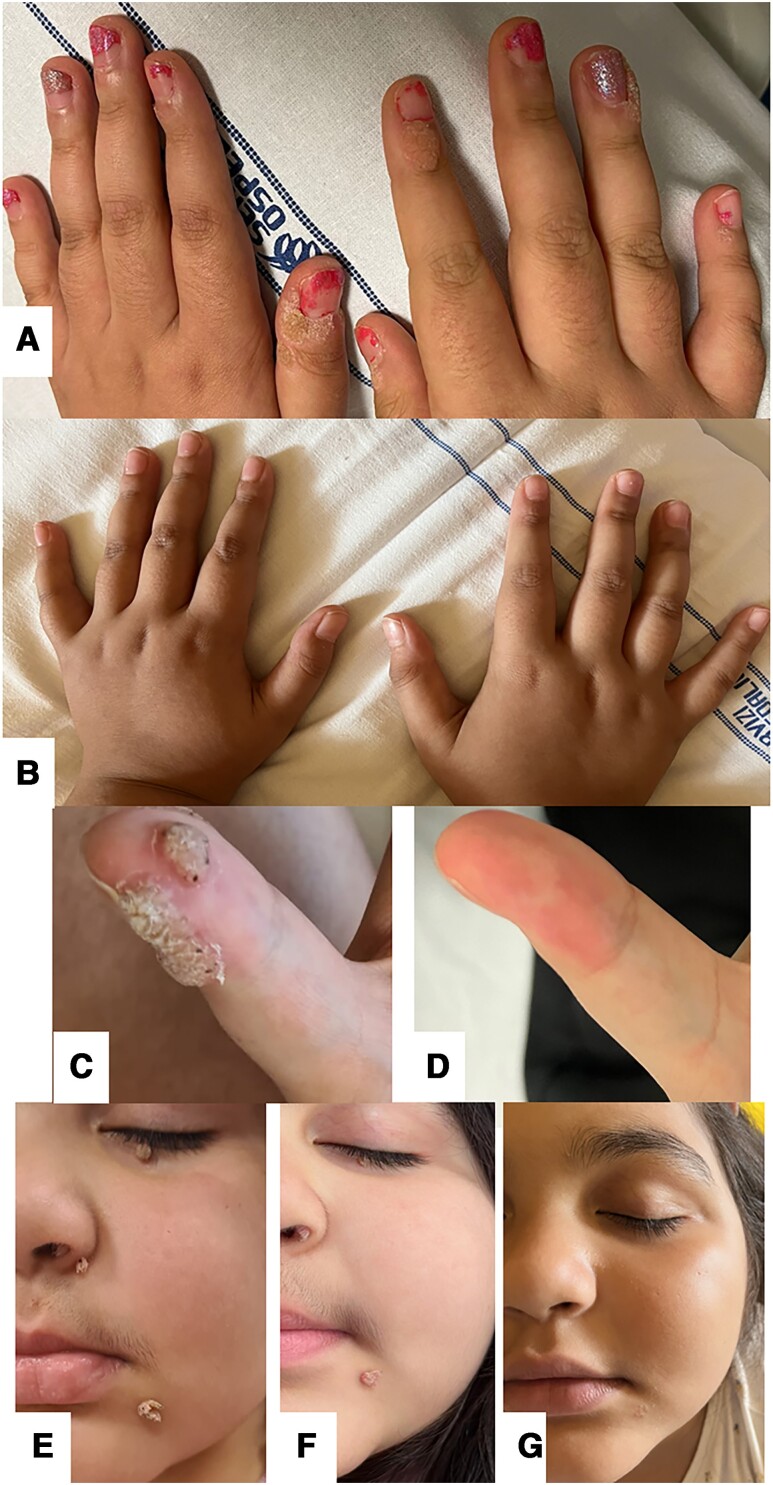
Finger warts before (*A*) and after (*B*) the vaccination. The thumb before (*C*) and after (*D*) the vaccination. Facial warts before (*E*) and after (*F*) the vaccination and after surgical removal (G).

A 9-year-old girl came to our attention for an immune-dysregulation disorder with disease onset at the age of 5 years, characterized by systemic juvenile idiopathic arthritis complicated by recurrent episodes of macrophage activation syndrome (MAS) and recurrent pulmonary infections. The patient had been treated for the past 4 years with multiple immunosuppressive treatments (intravenous steroids, tocilizumab, anakinra), with only partial disease control. Since the age of 7 years, she had presented also multiple recalcitrant CW and AW, recurring despite cryotherapy and electrocauterization. At clinical dermatologic examination, the patient presented facial warts, including intranasal, as well as digital periungual, and anogenital warts (Figure 1  *A*, *C*, *E*). At the age of 8 years, she presented with digital clubbing, with the appearance of bronchiectasias and mild interstitial lung disease on chest computed tomography. When out of the acute phases, she displayed normal leukocyte count, lymphocytic subsets, immunoglobulin levels, blood lymphocyte proliferation and natural killer cell perforin expression test. During flares, she presented very high serum interleukin 18 (IL-18) levels, whereas interferon signature was negative. A genetic panel for immunodeficiencies and type I interferonopathies was negative. The presence of a systemic juvenile idiopathic arthritis with recurrent MAS, extremely elevated IL-18, and pulmonary involvement is reminiscent of a recently described condition, named IL-18−associated pulmonary alveolar proteinosis and MAS syndrome, not linked to a definitive genetic mutation [23]. Because of the repeated MAS episodes, she was treated with high-dose steroids, canakinumab 4 mg/kg/month, cyclosporin 3 mg/kg/day, and baricitinib 4 mg/day. Contemporarily, warts were treated with multiple topical therapies (salicylic acid, 5-fluorouracil, cidofovir) without efficacy. A trial of baricitinib dosage reduction to 2 mg/day and steroids tapering for 6 months did not result in clinical benefit on the warts. Eventually, in line with a few positive case reports available in the literature, HPV-9V vaccination was administered. After 1 month from the first vaccine dose, a complete resolution of anogenital lesions and an almost-complete response of periungual warts (only 1 lesion persisted, reduced in size) was observed. Facial lesions only slightly reduced in size and were therefore removed with surgical shave removal after 3 months from the first dose, without any recurrence (Figure 1*E*-*G*). The histology showed a negative p16 staining. Even if a genotyping was attempted, it was not possible to isolate the specific HPV types. The patient underwent a second vaccine's dose after 6 months that led to the complete clearing of the residual hand warts (Figure 1*A*-*D*). So far, no wart relapses have been observed (currently at 12 months follow up).

## LITERATURE REVIEW

Per inclusion criteria, 28 articles were included. No randomized clinical trials were found on the topic, but mainly case reports and case series. In Supplementary Table 1, all the included articles with the warts' type, previous treatments, and response to vaccination are reported in detail.

### Immunocompetent Children

In children, the HPV vaccination seems to play a relevant role in contrasting CW. Indeed, of the 28 reported children with CW treated with the HPV vaccine, a complete remission was observed in 26/28 patients, with a partial remission in the other 2 [24–30]. As for AW, in children, 3 cases have been reported so far: Couselo-Rodríguez et al. treated a child with AW with 9V, obtaining a partial remission [31], whereas Kreuter et al. reported 2 children that showed an absent response to 4V [32].

In this count, the works by Yang et al. and Shin et al. [33, 34] were not included, dividing the patients in age ranges that did not allow to identify pediatric patients. However, both Authors reported a better response in the younger groups of patients: Yang et al. reported a complete response in 52% of the patients aged <20 years versus 38% in older patients [33], and Shin et al. reported a complete response in 84% of the patients aged 9–26 years, whereas only in 55% of the older patient group [34].

### Immunocompetent Adults

In healthy adults, a complete remission of CW after administration of the 2V was found in 14 patients, with a partial response in 6 patients. Interestingly, in the same study, a group of 22 patients treated with intralesional vaccine injections showed an higher response (complete response in 18 patients, partial in 2) [35]. With the 4V vaccination, a complete remission was reported in 9 cases [28, 29, 33, 36, 37], whereas with the 9V, it was reported in 28 of 58 patients [30, 34, 38–40] (Supplementary Table 1).

In adults’ AWs, a complete remission was observed in 7/15 patients after the administration of the 4V [41, 42] and in 3/6 after the 9V [43, 44]. Kreuter et al. reported instead a complete recurrence of AW in all the patients (n = 4) after treatment with electrocautery and 4V [32].

Also, 1 case of oral squamous cell HPV-positive papilloma, successfully treated with 4V is reported [45].

### Immunosuppressed Patients

In Table 1, the available literature data regarding the efficacy of HPV vaccination on clinically evident benign epithelial proliferations in immunosuppressed patients (adults and children) are reported [39, 40, 46–51].

**Table 1.** Characteristics of the Immunosuppressed Patients and Response to the HPV Vaccination

Author	No. of Patients	Underlying Disease	Immunosuppressive Therapies	Wart Type	Site	HPV Genotype	Vaccine	Response
Smith [51]	1 child	Neutrolymphopenia	0	CW	Hands, feet	NR	4V	C
Silling [52]	1	B-cell lymphatic leukemia → breast cancer	Chemo + radiotherapy, steroids, splenectomy	CW	Forearms, hands	HPVXS2	4V	C
Ferguson [48]	1	Ulcerative colitis	6MPU + mesalazine	CW	Trunk, extremities	NR	9V	P
Waldman [40]	2	P1 renal transplantation	NR	CW (flat)	P1: Trunk, extremities	NR	9V	NR (1 dead, 1 lost to follow up)
		P2 T-cell immunodeficiency			P2: Disseminated			
Tassavor [47]	1	HIV	HIV treatment (NR)	CW	Palmoplantar	NR	9V	P plantar
								C palmar
Bossart [39]	4	1 liver transplantation	CsA	CW	1 plantar	NR	9V	P
			Tacrolimus/CsA+		Fingers/fingers + plantar			
		2 kidney transplantations	MMF + PDN					
		1 CVID	MMF/AZA + PDN		Fingers + palms			
Moscato [49]	1	Severe B-cell reduction	0	CW + AW + CIN	Plantar + disseminated genital condylomas	CW: HPV-6	4V	C: CW
						Cervix: HPV-18, -33		A: AW + CIN
Kreuter [50]	1	WILD syndrome	NR	CW + AW	Disseminated	AW: HPV-6, -51, -52, -61, -84	4V	P CW
								A AW
						CW:HPV-57		

Abbreviations: 2V, bivalent HPV vaccine; 4V, quadrivalent HPV vaccine; AW, anogenital warts; AZA, azathioprine; C, complete; CIN, cervical intra-epithelial neoplasia; CNK, canakinumab; CsA, cyclosporine A; CVID, common variable immunodeficiency; CW, cutaneous warts; MMF, mofetil mycophenolate; NR, not reported; P, partial; PDN, prednisone.

In children, a treatment with the HPV vaccine is reported only in 1 case [51], who presented a complete response to the 4V on CW. In our case, the 9V was efficacious in treating the genital and perianal warts only a few weeks after the administrations of the first dose, with a complete response also on palmar and digital CW after the second dose, even if the face warts did not respond to the vaccination.

In adults, of the 9 treated patients presenting with CW, 1 patient presented a complete response with the 4V [46], whereas 6 patients presented a partial response to the 9V [39, 47, 48]. Two more patients were treated with the 9V, but the response could not be assessed (lost at follow-up, death) [40]. In the 2 patients presenting with both CW and AW, the vaccination was completely or partially effective on the CW, but was inefficacious on the AW [49, 50].

## DISCUSSION

Since first introduced in the late 2000s, the HPV vaccination rapidly established as successful primary prevention of anogenital cancer and AW, relevantly decreasing HPV infection rates [53, 54]. Yet, little is known about its effects on established HPV infections and related epithelial proliferations. Indeed, a potential therapeutic role of the HPV vaccination could be of great relevance, especially in difficult-to-treat patients, such as immunosuppressed patients, who are prone to develop generalized and persistent HPV infections even with LR-HPV types, possibly resulting in multiple proliferative recalcitrant epithelial tumors, such as mucous membranes papillomas and recalcitrant skin warts [55, 56].

The immune response to HPV infections is characterized by (1) a local intrinsic keratinocyte response and (2) by cellular and humoral activations [57].

The keratinocyte-intrinsic viral restriction is responsible for the asymptomatic viral clearance in immunocompetent patients, and when altered, can result in inborn errors of immunity such as epidermodysplasia verruciformis [58–60]. In this regard, it is of interest that although severe combined immunodeficiency (SCID) patients with RAG1 or RAG2 deficiency do not develop verrucosis after hematopoietic stem cell transplantation, those with IL2RG or JAK3 deficiencies might instead develop diffused HPV infections despite successful hematopoietic stem cell transplantation [61–63], possibly reflecting a keratinocytic intrinsic defect [57].

The cellular response is prevalently characterized by a CD4-mediated response, as highlighted by the development of diffused verrucosis in those with HIV or with inborn errors of immunity causing an elective CD4-T cell or antigen presenting cells (APC) impairment [52, 64]. Therefore, persistent HPV infections may be caused by an impaired keratinocyte response, allowing an HPV local replication and/or by an incapacity of APC and T cells to clear and control the infection [57].

The exact mechanism influencing the therapeutic effect of HPV vaccines has not yet been fully understood. Although the immune response to HPV infection is usually slow and there is no local inflammation, the efficacy of the available HPV vaccines is based on their ability to induce a humoral and cellular immune response. The HPV vaccines induce a robust antibody response against oncogenic HPV vaccine types and stimulate interferon-γ producing CD4+ and CD8 + T lymphocytes, effective in clearing and preventing the viral infection [65–67]. This cellular activation is followed by increased levels of tumor necrosis factor-α, IL-2, and proinflammatory cytokines (IL-1α, IL-1β, IL-6) [66]. These local alterations of cytokine microenvironments might play an additional role in the clearance of HPV-persistent infection and HPV-induced proliferations [65].

Our literature review has highlighted some positive responses to the HPV vaccination on benign intraepithelial proliferations, especially on CW and in younger patients. Young patients seem to generate higher numbers of HPV-specific memory B and T cells after HPV vaccine administration, thus leading to an enhanced immune response [68]. At the same time, a relationship between estrogen-related signalling and HPV-positive cancers has been demonstrated [69]. This might explain the reported age-dependent reduction of therapeutic vaccine effect [30, 33, 34] and the overall good response in children [24–29, 51], including our case report.

For immunocompetent adults, however, the efficacy remains controverted. In fact, from our literature review, only half of patients presented a complete response, which is approximately the same result that can be obtained with the standard topical or physical therapies [70]. Interestingly, higher response rates were obtained with intralesional vaccine injections [35, 71, 72], suggesting an important role of the keratinocyte-intrinsic viral response and of the local immune activation.

As far as immunosuppressed patients, few cases are reported, even if with encouraging results on CW (complete or partial responses reported). A different response, however, was observed in patients presenting both CW and AW, the latter showing always a minor/absent response to the vaccination [49, 50]. Yet, because immunosuppressed patients (especially HIV+ or having undergone renal transplantation) are more predisposed to generalized and persistent HPV infections [55, 56], the therapeutic use of HPV vaccination could represent an important tool in the control of HPV-related benign proliferations in these patients.

Still, very little is known about the ability of the available vaccines to cross-react on different HPV types. It has been postulated that a cross-reactive immune response is elicited by the vaccine depending on the phylogenetic similarities between L1 genes in the different HPVs (HPV-16 with HPV-31, 33, 52, 58, and HPV-18 with HPV 45) [1, 73, 74], with several studies reporting a cross-reactive response induced by the 2V and 4V against the aforementioned genotypes [14, 65, 75–77]. Also, differences in cross-protection could depend on different adjuvant systems [73]. Interestingly, in some cases reported in this review, different HPV types responding to 2V or 4V were reported [9, 24, 38, 45, 46, 49]. Our patient treated with the 9V, presented a complete resolution of AW and of the finger lesions, whereas the facial warts only slightly responded. Even if a genotyping was attempted, it was not possible to isolate the specific HPV types. However, such a different response seems to underlie different HPV-type infections.

This literature review has some important limitations. First, the reported data are based on case reports and case series, with no clinical trials reported on the topic. Second, there is the possibility of a publication bias because mainly successful cases are reported in the literature. Also, it should be kept in mind that the use of the HPV vaccine for treatment is off-label and should not be considered as standard therapy.

In conclusion, we report the case of an immunosuppressed young girl with recurrent recalcitrant warts that presented a good clinical response to the HPV vaccine. We reviewed the existing literature on therapeutic HPV vaccination. We conclude that larger, systematic case studies on the therapeutic effects of HPV vaccination are needed to understand its efficacy on different lesions and populations and the immune mechanisms behind it.

## Supplementary Material

ofae369_Supplementary_Data

## Supplementary Data

Supplementary materials are available at *Open Forum Infectious Diseases* online. Consisting of data provided by the authors to benefit the reader, the posted materials are not copyedited and are the sole responsibility of the authors, so questions or comments should be addressed to the corresponding author.

## Notes

***Financial support*.** The authors declare no financial support for this paper.

***Patient consent statement***. Complete written informed consent was obtained from the patient for the publication of this study and accompanying images.

Data sharing is not applicable to this article as no datasets were generated or analyzed during the current study. All the included articles can be found in Supplementary Table 1.
