# Supplementary material for: Therapeutic Role of HPV Vaccination on Benign HPV-induced Epithelial Proliferations in Immunocompetent and Immunocompromised Patients: Case Study and Review of the Literature
Source: Open Forum Infect Dis. 2024 Jul 19;11(7):ofae369. doi: 10.1093/ofid/ofae369 (PMC11259138; doi:10.1093/ofid/ofae369)
Supplement: ofae369_Supplementary_Data [file ofae369_supplementary_data.docx]

**Supplementary Table 1.** Literature review about the effects of HPV vaccination on benign HPV-induced epithelial proliferations.

| **Author** | **N° pts** | **Adults/**  **children** | **Warts’ type** | **Location (number)** | **Immunosuppression** | **Sex** | **Age** | **Vaccine type** | **Response** | **Previous therapies** | **HPV genotype** |
| --- | --- | --- | --- | --- | --- | --- | --- | --- | --- | --- | --- |
| Landini (24) | 1 | Ch | CW | Plantar, Hands (multiple) | No | M | 16 | 4V, 3 doses | C | Kera, ECT, cryo, photo, IMI, IV cidofovir | HPV-2 |
| Abeck (27) | 6 | Ch | CW | Palmar/Plantar (multiple) | No | 2M,  4F | 2pts: 9  3 pts: 10  1 pt: 11 | 4V, 3 doses | C | IMI (3), laser (2), cimet (1), 5-FU (5), glutaraldehyde (5) | NR |
| Martin (25) | 5 | Ch | CW | Hands, elbows, knees, trunk | No | F | 9, 10,12,13,14 | 4pt: 2V 2-3 doses; 1pt: 4V 3 doses | C | Cryo, SA | NR |
| Smith (51) | 1 | Ch | CW | Hands, Feet (multiple) | Yes | M | 17 | 4V, 3 doses | C | Cryo, IMI, Kera | NR |
| Martin (26) | 1 | Ch | CW | Finger (1) | No | F | 10 | 2V, 1 dose | C | Cryo, SA, cantharidin | NR |
| Couselo- Rodriguez (31) | 1 | Ch | AW | Vulvar, exophytic | No | F | 11 | 9V, 2 doses | P | IMI, laser | Inhibited sample |
| Hayashi (29) | 3 | 2 Ad,  1 Ch | CW | P1: hands/forearms (80)  P2: hands,toes (30)  P3: plantar (1) | No | M | P1: 70  P2: 9  P3: 65 | 4V,  4 doses (Ad)  3 doses (Ch) | C 66,6% (P1-2)  A 33,3% (P3) | Cryo, ASA, IMI, cimet | HPV-27 (Ch), HPV-57 (Ad) |
| Daniel (28) | 4 | 1 Ad  3Ch | CW | Face, Hands, wrists,  elbows, knees,  feet | No^ | 2M, 2F | 5, 6, 8, 20 | 4V; 3 doses | C | 2 naïve, Cryo (2), cimet (1), IMI (1), SA(1) | NR |
| Shin MK (30) | 34 | 23 Ad 11 Ch | CW | NR | No | M & F | 11 pt <18y  11pt 20-30y  12 pt>30y | 9V | <18yrs:  C 81.8%  P 18.2%  20-30y:  C 45.4%  >30y: C33% | NR | NR |
| Yang (33) | 30 | Ad, Ch | CW |  | No | 11M,  19F | 6-55 (mean 21.4 yrs) | 4V,  3 doses | C 46.7%  P 16.7%  A 36.7%  <20 y  C 45%  P 10  A 30  >20 y  C 38%  P 23 %  A 38% | Naïve | NR |
| Shin JO (34) | 45 | Ad&Ch, | CW | Plantar, Periungueal | No | 22M, 23 F | 25pts <26y  20pts >26y | 9V,  3 doses | C 62.2%  P 8.9%  A 28.9%  [9-26 years  C 84%  >26 years  C (55.0%)] | Cryo, SA, IMI, laser, 5FU, cimet, IL-MMR/Bleo, surgery, ECT, kera | NR |
| Venugopal (36) | 1 | Ad | CW | Hands (multiple) | No | M | 31 | 4V; 3 doses | C | podo, SA, Cimet, Cryo, IMI | NR |
| Landis (37) | 1 | Ad | CW | Feet (4) | No | F | 59 | 4V; | C 100% | cryo, topical tretinoin, SA, IL bleo | Positive HPV probe for in situ hybridization |
| Silling (46) | 1 | Ad | CW | Forearms, backs of hands, fingers (multiple) | Yes | F | 41 | 4V; 3 doses | C | SA, podo, 5-FU, IMI, cryosurgery, curettage, ECT, laser | HPV-XS2 |
| Ferguson (48) | 1 | Ad | CW | Trunk, extremitiess (>70) | Yes | M | 77 | 9V, 3 doses | P | LN2, SA, shave removal | NR |
| Waldman (40) | 16 | Ad | CW | Fingers, hands | Yes (2);  No (14) | 15 M, 1 F | 4: 18–35 y  6: 36–50 y  6: 51–80 y | 9V, 3 doses | C 43.7%  A 37.5% Lost at f-up: 18.7%  Dead: 6% | LN2, candida, IMI, curettage, SA, cimet, 5FU, cidofovir, PDL | NR |
| Bossart (39) | 5 | Ad | CW | Fingers, palmar, plantar | 4 yes  1 no | 3M, 2F | 19-65 | 9V, 3 doses | C 20%  P 80% (1 immunosuppressed) | podo, IMI, green tea extracts, Cryo, CO2 laser ablation | NR |
| Nofal (35) | 44 | Ad | CW | Hands, feet, forearm, knee, face | No | IM:  12M, 10 F  IL:  12M 10 F | IM: 17-54y  IL: 16-45y | 2V IM, 3 doses  2V IL: every 2 weeks, max 6 injections | - 22 IM**:**  C 63.3%  P 27.3%,  A 9.1%  - 22 IL:  C 81.8%,  P 9.1%,  A 9.1% | Kera, cryo, ECT, surgical, MMR | NR |
| Tassavor (47) | 1 | Ad | CW | Plantar (>21), thumb (1) | **Yes** | M | 56 | 9V | P foot  C thumb | Cryo, ECT, IMI, IL candida & bleo |  |
| Kazlouskaya (38) | 1 | Ad | CW | pubis, inner  thighs, and left inguinal fold (multiple) | No | F | 79 | 9V; 1 dose | C | IMI | HPV 16, 18, 6, 11 |
| Dianzani (43) | 1 | Ad | AW | Perianal | No | M | 65 | 9V; 3 doses | C 100% | IMI | NR |
| Kreuter (32) | 6 | 4Ad 2Ch | AW | 1 pt genital, 2 pts genital+perianal, 2 ptss perianal, 1pt perianal+intra-anal | No | 5M, 1F | 12,13,19, 34, 37,42 | 4V, 3 doses | A: recurrence 100% | ECT (1 pt laser, IMI, podo) | 1pt HPV-57, 4 pt HPV-6 1 pt HPV- 6+55 |
| Moscato (49) | 1 | Ad | CW + AW  + CIN | Plantar +  disseminated genital condylomas | Yes | 24 | F | 4V; 3 doses | C CW  A AW | Laser, IMI, retinoid acid derivatives | Cervix: HPV-18, 33  CW: HPV-6 |
| Kreuter (50) | 1 | Ad | CW + AW | Disseminated (multiple) | Yes | 41 | F | 4V; 3 doses | P CW  A AW | NR | AW: HPV-6,51,52,61,84;  CW: HPV-57 |
| Choi (41) | 10 | Ad | AW | Genital condyloma acuminata (1 pt: 1; 5 pts 2-4; 4pts >5) | No | 9M, 1F | Mena age 26.2±6.0 | 4V; 3 doses | C 60%,  P 30%  A 10% | 14 naïve  Cryo, ECT, laser | NR |
| Lee (42) | 1 | Ad | AW | Perianal | No (diabetes) | M | 46 | 4V; 3 doses | C 100% | IMI | NR |
| Bossart (44) | 5 | Ad | AW | 4 pts penile, 1 pt groin and  perineum | No | 5M | 21-58 | 9V; 3 doses | C 40%,  P 60% | IMI, laser, cryo | NR |
| Cyrus (45) | 1 | Ad | Oral squamous cell papilloma | lips, tongue, and buccal mucosa | No | M | 60 | 4V; 3 doses | C | Surgical excision | HPV-32 |

Ad= Adult ≥ 18 years; Ch= children <18 years, AW Anogenital Warts, CW cutaneous Warts, C =complete response, P partial response, A= Absent response, IM= intramuscular, IL= intra-lesional, NR =not reported; 2V bivalent vaccine, 4V quadrivalent vaccine, 9V nonavalent vaccine; cryo, cryotherapy; IMI, imiquimod, Kera, keratolytics; Cimet, cimetidine; ECT electrocautery, SA salicylic acid; photo, photodynamic therapy; Bleo, bleomycin, LN2 liquid nitrogen, podo, podophyllotoxin

^1 Ad on steroids occasionally for granuloma annulare
